# Investigation of Fractal Carbon Nanotube Networks for Biophilic Neural Sensing Applications

**DOI:** 10.3390/nano11030636

**Published:** 2021-03-04

**Authors:** Leo A. Browning, William Watterson, Erica Happe, Savannah Silva, Roberto Abril Valenzuela, Julian Smith, Marissa P. Dierkes, Richard P. Taylor, Natalie O. V. Plank, Colleen A. Marlow

**Affiliations:** 1 School of Chemical and Physical Sciences, Victoria University of Wellington, Wellington 6021, New Zealand; leo.browning@vuw.ac.nz (L.A.B.); erica.happe@vuw.ac.nz (E.H.); natalie.plank@vuw.ac.nz (N.O.V.P.); 2 The MacDiarmid Institute for Advanced Materials and Nanotechnology, Victoria University of Wellington, Wellington 6021, New Zealand; 3 Materials Science Institute, Physics Department, University of Oregon, Eugene, OR 97403-1274, USA; william.j.watterson@gmail.com (W.W.); jsmith767@gmail.com (J.S.); rpt@uoregon.edu (R.P.T.); 4 Physics Department, California Polytechnic State University, San Luis Obispo, CA 93407, USA; ssilva22@calpoly.edu (S.S.); rabrilvalenzuela@physics.ucsb.edu (R.A.V.); marissadierkes@gmail.com (M.P.D.)

**Keywords:** carbon nanotubes, fractal networks, neural sensing, non-linear processes

## Abstract

We propose a carbon-nanotube-based neural sensor designed to exploit the electrical sensitivity of an inhomogeneous fractal network of conducting channels. This network forms the active layer of a multi-electrode field effect transistor that in future applications will be gated by the electrical potential associated with neuronal signals. Using a combination of simulated and fabricated networks, we show that thin films of randomly-arranged carbon nanotubes (CNTs) self-assemble into a network featuring statistical fractal characteristics. The extent to which the network’s non-linear responses will generate a superior detection of the neuron’s signal is expected to depend on both the CNT electrical properties and the geometric properties of the assembled network. We therefore perform exploratory experiments that use metallic gates to mimic the potentials generated by neurons. We demonstrate that the fractal scaling properties of the network, along with their intrinsic asymmetry, generate electrical signatures that depend on the potential’s location. We discuss how these properties can be exploited for future neural sensors.

## 1. Introduction

Fractal patterns have been observed in electrical currents as they spread out through mesoscopic systems [1] and also in how the conduction varies when electric and magnetic fields are applied [2,3]. Fractals are prevalent in nature, and their repetition of patterns at different magnifications generates significant complexity. Investigations of this complexity have broadened our understanding of fundamental aspects of electrical conduction mechanisms. In addition, exploiting the associated non-linear response of fractals has the potential for novel applications. Investigations of fractal conduction effects induced by electrons scattering off device walls and material impurities [1,2,3,4] inspired simulations of conduction through fractal circuits. The proposed practical examples of fractal circuits include solar panels [5], retinal implants [6,7], and transistors [8]. For the latter case, electrostatic gates were used to deplete regions of the circuit, and the subsequent re-routing of current displayed highly non-linear changes in the conduction. The electric field generated by neurons firing should in principle also induce non-linear changes in the conductance of fractal circuits. Here, we propose applying this effect for neural sensing.

The ability to record signals from large numbers of neurons simultaneously in the intact brain represents a major interdisciplinary challenge. An ideal sensor needs sensitivity and spatial accuracy to precisely track the electrical activity of multiple neurons over time combined with non-subjective measures of signals with quantified errors and minimal post processing. Current applications of carbon nanotube (CNT) thin films in neural sensing use CNT thin films in the metallic phase to replace the electrodes used to stimulate and record neural activity [9,10]. While these applications promise to improve the electrode performance by exploiting the exceptional properties of CNTs to create flexible, highly conductive interfaces with a large surface area, they build on conventional in-vivo electrical recording strategies. Conventional in-vivo electrical recordings use extracellular probes that are limited by signal variability amongst neurons, electrical drift, and post-processing using algorithms [11,12]. Here, we propose a field effect transistor (FET) sensor that exploits the inherent sensitivity of a fractal network of conductors and has the potential to overcome these limitations. By employing CNT films not as electrodes but rather as the active layer in the FET, we open up the opportunity for a distinctly different operational design for multi-neuronal sensing. The proposed sensor features 16 electrodes connected to different locations along the network’s perimeter. The simultaneous measurement of multiple currents can then be used to detect neuron-induced changes to conduction in the network occurring due to the electric field effect. For sensor operation, the material chosen for the fractal network must also have a measurable gating response to typical electric fields generated by neurons firing, and for simultaneous recording with spatial accuracy it must lack inhomogeneity to exhibit detectable electrical asymmetries across the network. Additionally, the selected active layer needs to be biocompatible with neurons. We will present experimental results which demonstrate that thin films of randomly aligned carbon nanotubes have all these necessary characteristics for our neural sensors.

Previous studies have shown that neurons prefer to adhere to CNT structures [10,13,14] rather than to the smooth surfaces of traditional sensors [10]. Thin film CNT FETs have been employed for a range of electronic sensing applications, from hormone detection to the detection of molecular dynamics [15,16,17,18,19]. CNT thin films are also known to form complex networks with morphologies that affect their FET performance [16,19,20,21,22,23]. However, although this complexity makes them a compelling candidate for the fractal active layer of our sensors, to our knowledge the fractal aspect of CNT thin films has not been verified until now. We will use fractal dimension, *D*, to quantify the relative contributions of the fractal network’s coarse and fine structures to its conduction properties. We will also demonstrate that the ability to change the CNT thin film’s density can be used to tune the network’s *D* value and so impact the network’s electrical sensitivity.

Within this picture, how the current percolates through the fractal network is crucial. To conduct current, a CNT network must feature at least one continuous path between electrodes. Below a certain CNT density (the percolation threshold, *ρ_th_*), the network has zero probability of forming a continuous path, and the network’s properties vary drastically in proximity to this critical density. As a result, CNT thin films exhibit significant electrical sensitivity near *ρ_th_*. We will therefore map the dependence of the CNT network’s electrical sensitivity as a function of its *D* value in order to determine the optimal percolation conditions for the neural sensor.

We first use simulations to demonstrate the operational principles of our sensor design and to establish the relationship between the percolation and fractal dimension of simulated CNT networks. Then we use atomic force microscopy (AFM) to compare the fractal nature of CNT thin film samples to simulations. Next, we present electrical characterization measurements from two device designs, one to investigate inhomogeneity using conduction measurements between multiple electrodes and the second to investigate the network’s response to electric fields. The latter uses SU-8 encapsulated top gates to generate electric potentials similar to neurons. Finally, we will return to simulations for a systematic, controlled study to understand the relationship between network sensitivity and *D*. Taken together, this interplay of simulations and experimental measurements demonstrates the promise for future studies of CNT-based fractal sensing of neural networks.

## 2. Materials and Methods

### 2.1. Sensor Simulations

To illustrate the operational principles of our sensor we simulated the current through a 16 electrode FET in response to multiple neuron scenarios. Each scenario varied in number of neurons (1 or 2), location of neurons (*x* and *y* position within the plane of the channel and height above the surface (0 or 15 μm)), and the magnitude of the neuron’s signal (magnitude of electric potential). For simplicity, we used a uniform semiconducting channel that, when depleted, undergoes a change in resistance proportional to the surface potential responsible for the depletion [8]. We obtained the voltage profile at the channel surface due to the electric field of a neuron firing by assuming neurons are point sources at their respective locations. Lastly, the current through pairs of electrodes spanning the channel was calculated for each scenario.

### 2.2. CNT Network Simulations

CNT thin film networks were modeled using a 2-dimensional (2-d) random stick network and the Monte Carlo process. CNTs were represented as sticks with a fixed length. The tube length and overall size of the network were chosen to be consistent with the observed CNT network morphology determined from AFM and typical device channel sizes. The sticks were assigned to be either metallic or semiconducting and placed at random *x*-*y* positions and orientation angle in the simulated device channel. A black and white bitmap image was created for each simulated network and a fractal analysis performed on its structure.

We also simulated the impact of the electrostatic gating for a subset of networks to understand the role of network structure in electrical sensitivity. CNT networks are junction-dominated networks, meaning that the network resistance is dominated by the barriers that form at the junctions between tubes [24,25]. Therefore, to simulate the electrical properties of the network we included only the contributions of the inter-tube junctions. The simulated network was simplified using graph theory. A node represented each tube, and the junctions between two overlapping sticks were represented as edges. Modified nodal analysis was used next to solve Kirchhoff’s equations and calculate the network current. Junctions between metallic-metallic (m-m) tubes and semiconducting-semiconducting (s-s) tubes were assigned resistances of 100 kΩ and 1 MΩ, respectively, based on typical junction resistances measured experimentally [23,24]. While m-m and s-s junctions exhibited linear current voltage characteristics, metallic-semiconducting (m-s) junctions showed leaky diode characteristics thought to arise from the Schottky barriers [22,23]. Therefore, we approximated the current across m-s junctions using the ideal diode equation. An applied electrostatic gate will impact the m-s junction current by altering the size of the energetic barrier [25]. This effect was modeled by assuming a linear dependence of the junction barrier height on gate voltage. For each simulated gate voltage, the current through each m-s junction was determined using an adjusted barrier height. This value along with m-m and s-s junction resistances was then used to calculate the network current as a function of gate voltage, *V_g_*.

### 2.3. CNT Thin Film’s Deposition

The composition of the CNT thin film should be of high semiconducting tube purity in order to maximize the field effect response of the active layer for sensor operation. CNT thin films were fabricated with varying densities using solution deposition [21,26,27,28,29]. The CNTs used were purchased and pre-purified by NanoIntegris (IsoNanotubes-S Buckypaper composed of single-walled CNTs containing 99% semiconducting CNTs) [28]. A 5 μg/mL CNT dispersion was made from Buckypaper weighed on a precision balance (Sartorius ME36S, Sartorius AG, Göttingen, Germany) and dispersed into dichlorobenzene (DCB) (99% Sigma Aldrich, St. Louis, MO, USA) by an ultrasonic bath (Sonorex DT 100, Bandelin electronic GmbH & Co. KG, Berlin, Germany) with an average power of 80 Watts for 15 min. Degenerately doped Si with a 300 nm SiO_2_ layer was used as the device substrate. The substrates were prepared for CNT adhesion using a polydimethylsiloxane (PDMS) stamping method [26]. A blank cured PDMS stamp was made using Sylgard 184 (Dow Corning, Midland, MI, USA) with a base to curing agent ratio of 10:1, which was mixed and then degassed via vacuum desiccation and cured in air at room temperature in a plastic container lid. The stamp was then exposed to an O_2_ plasma (Plasma Etcher-50, 50 W, 20–50 KHz) (Plasma Etch, Carson City, NV, USA) for 1 min prior to spin coating a 10 mg/mL solution of 2-mercaptopyridine (Sigma Aldrich) at 2000 rpm with an initial acceleration of 500 rpm/s for 40 s. The 2-mercaptopyridine-coated PDMS was then pressed down on top of the clean substrate surface for 3 min followed by a 3 s rinsing in ethanol to remove any excess 2-mercaptopyridine. The substrates were then dried in a stream of N_2_, ensuring no remaining ethanol was present, and then submerged in the prepared CNT-DCB suspension for a fixed amount of time. Using our technique, the CNT network density can be controlled by varying the submersion time of the CNT deposition [21,27]. We used submersion times from 1–80 min, which yielded densities varying from 3.0 to 21.2 tubes/μm^2^. After CNT deposition the substrates were submerged in ethanol for 10 min and then blow-dried with N_2_.

### 2.4. Structural Characterization of CNT Thin Films

The characterization of CNT thin films’ morphology was performed using atomic force microscopy (Asylum MFP-3D, Santa Barbara, CA, USA). To determine the density, average tube length, and fractal geometry of the networks, AFM images were analyzed using the image analysis software ImageJ [30] (1.51, National Institutes of Health, Bethesda, MD, USA). Raman spectra were performed after CNT thin film deposition to verify that the tube diameter and purity agreed with the manufacturer’s specifications. The results are shown in Figure S1.

#### 2.4.1. CNT Network Morphology

It is common for CNT thin films produced via buckypaper deposition routes to be composed of both individual tubes and bundles of tubes [21,25]. In our analysis, both tubes and bundles of tubes were counted as tubes (both the equivalent of a stick in our simulated random stick network). The density and average tube length were determined from at least three AFM images from different 4 μm × 4 μm regions of the film. Tube density was determined by counting the number of tubes present in each image, and average tube length was determined from the measured length of all tubes and bundles. The density value reported is the average value determined from all the images from different regions of a given thin film. The uncertainty in this value is defined as one standard deviation of the value for each set of images.

#### 2.4.2. Fractal Analysis

Fractal analysis was performed using standard box-counting [31] using the FracLac feature of ImageJ. For each 7 × 3 mm thin film sample, several 10 μm × 10 μm high resolution images were taken at different locations on the CNT network film. For each simulated network, a black and white bitmap image was generated for analysis. However, to perform the analysis on the experimentally fabricated CNT networks, AFM images were processed to create black and white bitmap images. The sticks and individual CNTs were one pixel thick in the simulated and fabricated network images, respectively.

### 2.5. Device Fabrication

**Figure 1:**
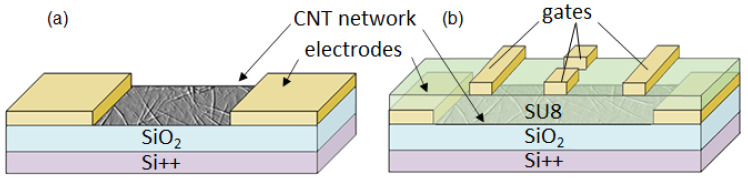
Schematics of the device designs: (**a**) an open channel carbon nanotube (CNT) network multi-electrode device and (**b**) an SU-8 encapsulated multi-top gate CNT network field effect transistor (FET).

The CNT devices were fabricated using standard photolithographic techniques. Schematics for the devices are shown in Figure 1, with (a) an open channel multi-electrode device and (b) a SU-8 encapsulated multiple-top gated FET. To pattern the CNT networks, the FET channel region was protected by AZ1518 (Microchemicals, Newton, MA, USA) photoresist, and the remaining exposed CNT film was etched by 3 min exposure to a 200 W O_2_ plasma (Oxford Instruments, Plasmalab 80 Plus, Abingdon, United Kingdom ). The photoresist mask was then removed by acetone and IPA cleaning. Source and drain electrodes were patterned via photolithography, in alignment with the FET channel region and deposited by thermal evaporation of Cr/Au 5/50 nm (Angstrom Engineering, Nex Dep 200, Kitchener, ON, Canada).

For the FET devices onto which localized top gates were fabricated (Figure 1b), the source and drain electrodes were patterned using AZ5214E (Microchemicals) photoresist. This change in resist was to prevent the formation of edge spikes on the electrodes. A 325 nm thick top dielectric was fabricated by dissolving GMPI-60 SU8 (Microchemicals) in cyclopentanone to produce a low viscosity solution, 15% by weight, which was spun onto the CNT FETs at 6000 rpm for 40 s. To uncover the source and drain electrodes, the SU8 was patterned using photolithography followed by development in an SU8 developer (Microchemicals). Top gates (thermal evaporation of Cr/Au 5/50 nm (Angstrom Engineering, Nex Dep 200)) were then fabricated over the dielectric layer via photolithography.

### 2.6. Electrical Measurements

All electrical measurements were taken at room temperature. Current and voltage measurements were taken using a Keithley sourcemeter (2400) (Solon, OH, USA). Electrostatic gating measurements were performed using an Agilent 4156C parameter analyzer (Santa Clara, CA, USA) on a Rucker and Kolls probe station (Milpitas, CA, USA). For the gating measurements, a source-drain voltage of 100 mV was applied while the individual gate voltage, *V_g_*, was swept from −20 V to 20 V. To quantify the electrical sensitivity of the device to a given gate, the normalized current change Δ*I*/*I*_0_ = (*I* − *I*_0_)/*I*_0_, was calculated from the resulting transfer characteristics. *I*_0_ is the network current with no applied gate, *V_g_* = 0.

## 3. Results

### 3.1. Simulation Results

#### 3.1.1. Sensor Architecture

**Figure 2:**
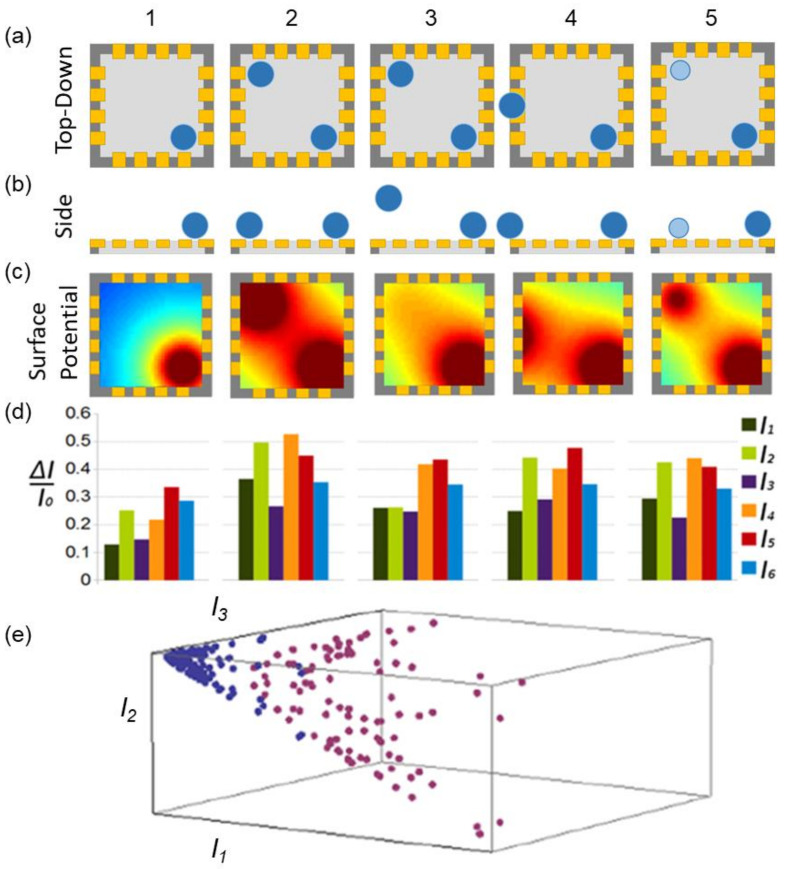
Illustrative examples of neurons (blue) acting as electrostatic gates on the active layer (light gray) of a multi-electrode FET probe using 16 electrodes (gold) to serve as source and drain. Five different neuronal positions and magnitudes, shown per column, lead to unique sets of current measurements. The top-down (**a**) and side projections (**b**) of 10 μm (dark blue) or 7 μm (light blue) neurons. (**c**) The unique surface potentials associated with the (*x*, *y*, *z*) coordinates of neurons in each case. (**d**) The associated normalized current changes, Δ*I*/*I*_0_ = (*I* − *I*_0_)/*I*_0_, through specific electrodes in each case. *I*_0_ is the electrode’s current with no stimulus. (**e**) The current signatures (for three currents) are shown for one neuron at different locations and signal strengths (purple = small; signal, magenta = large signal).

The fundamental operational principles of the multiple electrodes FET design are illustrated in Figure 2. The 16 electrodes arranged around the active layer allow for 15 independent measurements of the current in the channel. The simulations quantified the sensitivity of the 15 measurements to the location and magnitude of the electric field generated by neurons firing (their positions indicated by the blue circles in Figure 2a,b). Five example neuron scenarios, numbered 1–5, are shown by column in Figure 2. For each scenario, the resulting potentials at the sensor surface and the current between six terminals were calculated (Figure 2c,d, respectively). The six current measurements show the unique current signature for each of the neuron configurations. In particular, note that a weak neural signal in contact with the sensor’s surface (scenario 5) produced a different signature to a strong signal 15 μm above the surface (scenario 3). Consequently, the sensor can distinguish the signal’s origin (*x*, *y*, *z*) and magnitude for each neuron.

Current measurements were associated with unique values for position and size of each signal’s depletion pattern. The plot shown in Figure 2e was created using just three current measurements for one neuron at different locations and signal strengths. The library of data points plotted in the current space represents the range of different scenarios for a given neuron. In future applications, once a library is established, a search algorithm could then be used to convert measured current values into the positions and magnitudes for each neuron present. The accuracy of the sensor will depend on the degree to which scenarios can be distinguished in the library, and this is determined by how the data points fill the available space. In particular, degeneracies (i.e., two scenarios occupying the same data location) will increase if data points occupy only a small fraction of the current space.

The improvement of sensor performance can be achieved by increasing the number of electrodes, because the signature will then feature more distinguishing current components. Ultimately however, the fabrication capabilities will limit the size, and hence the number of possible electrodes. A more effective strategy for lifting degeneracy would be to replace the uniform channel with a statistical fractal network (i.e., a network in which the statistical characteristics of the patterns repeat at different scales). As in the uniform channel, a fractal network depletion of a local region of the channel will induce a re-arrangement of current. However, the non-linearity of this re-arrangement produces an enhanced sensitivity of the measured current to the location of the depletion region, and hence to the neuron signal. A statistical fractal takes advantage of the sensitivity to electrical changes that fractal conductors have been shown to exhibit [8] while also introducing asymmetry in the channel’s response. The inhomogeneity of a statistical fractal channel will lead to a lack of electrical symmetry that can reduce the locational degeneracy between neurons. Together, the non-linearity and asymmetry of the conductive layer will increase the spread of data in the current space, increasing our ability to distinguish between the unique current signatures of different neuronal scenarios.

#### 3.1.2. CNT Network Structure

**Figure 3:**
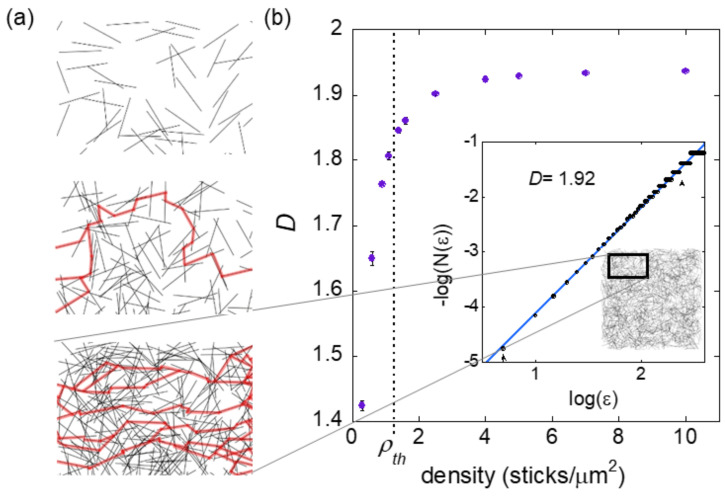
(**a**) Details of simulated networks at densities of 0.6, 1.4, and 4.0 (top to bottom) with any percolated paths highlight in red. (**b**) Fractal dimension, *D*, of simulated CNT thin film networks as a function of density. The *D* value changes rapidly near the percolation threshold (*ρ_th_* = 1.172 sticks/μm^2^) indicated with a dashed line. Error bars indicate the variance of *D* value for three networks at each density. The inset shows the box-counting plot resulting from fractal analysis for a network with a density of 4.0 sticks/mm^2^. The linearity of the plot indicates fractal scaling with the slope of the best-fit line, which determines the fractal dimension *D* (value given for plot shown).

Having established the basic operational principle of the sensor, the next step in our investigation was to use simulations to confirm that randomly-aligned CNT thin films form networks with statistical fractal characteristics. If statistically fractal, they will satisfy the two essential elements for the sensor discussed above (the non-linear sensitivity and inhomogeneity). However, it is also important to understand how proximity to percolation plays a role in the operation of the neuron sensor. Previous work has shown the enhanced electrical sensitivity of CNT thin films close to their percolation threshold [20,21]. To investigate how the fractal structure evolves near percolation, we simulated CNT films for a range of densities straddling the percolation threshold. Our results are shown in Figure 3.

Networks with dimensions of 20 μm × 20 μm and tube length of 2 μm were simulated at 11 different densities (three networks per density), and fractal analysis was performed on the resulting networks to determine their *D* values. A tube length of 2 μm was chosen to be consistent with the actual CNT thin films. The densities ranged from 0.3 to 10 sticks/μm^2^ and spanned the percolation threshold for this size network of 1.172 sticks/μm^2^. The percolation threshold was determined by generating 100 networks and using percolation theory to fit a plot of the network’s probability of reaching percolation versus its density. Figure 3a shows 9 μm × 6 μm regions of three example networks with different densities.

Our analysis shows that the simulated networks display fractal characteristics. We used the traditional form of fractal analysis in which the image is covered with a mesh of identical squares (boxes). For a network to be considered fractal, the minimum number of boxes, *N(ε)*, required to cover the network scales according to box size, *ε*, as *N(ε)~ ε^−D^*. As an example, the box-counting plot for a network with a density of 4 sticks/μm^2^ is shown in the inset to Figure 3b. The linearity of the plot indicates that the simulated networks are fractal within the observable range indicated by the arrows. The lower limit of observation was set by the smallest feature size (the thickness of a tube, 1 pixel) and the upper limit set by the counting statistics of the box-counting algorithm (20% of the total network size). The fractal dimension for each case was determined from the slope of the best-fit line.

Figure 3b shows the average *D* at each density for all networks and reveals a clear dependence of *D* on density. Near the percolation threshold (dashed line in Figure 3b), *D* changes rapidly and then saturates at higher densities. We also see the largest variance in *D* value for networks of the same density near percolation. When interpreting the shape of this curve, recall that *D* is an indicator of the ratio of fine to course structure of the network. As more tubes are added to the network, its fine structure increases, raising the *D* value. Although fractal analysis has not been previously applied to determine the structure of the network composing CNT thin films, studies of the surface roughness of CNT coatings determined CNT film surfaces to be bi-fractal (quantified by one *D* value across micro-length scales and another *D* across nano-length scales) [32]. DeNicola et al. found that at densities well above percolation the surfaces had the same *D* values for all densities [32] consistent with our results.

Our results reveal a saturation in *D* value—a signature of the network moving past the percolation threshold predicted by percolation theory. For a CNT network, how tubes fill the 2-d space of the channel is intimately connected to the formation of paths and the likelihood of percolation. *D* is a quantified measure of how the tubes fill space and therefore an appropriate indicator of the evolution of a network through percolation. In Figure 3a we show details of three simulated networks with densities below, just past, and well above the percolation threshold. These illustrate how the structure of a CNT network evolves when adding more tubes to the network. Below the percolation threshold, the network is not connected. Just past the threshold, few conductive paths have formed, and this therefore offers few detour possibilities for bypassing a given region. Further past the percolation threshold, many paths are established, and the addition of more tubes increases the number of interconnects between them rather than significantly increasing the path number.

### 3.2. Experiemental Results

#### 3.2.1. Fractal Analysis of CNT Network Thin Films

**Figure 4:**
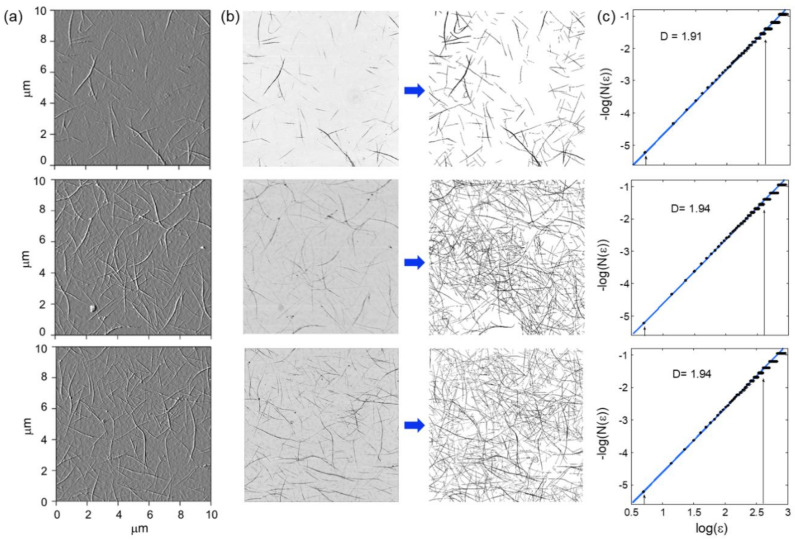
Fractal analysis of a random CNT thin film network structure. (**a**) Atomic force microscopy (AFM) images of typical CNT thin film networks (note the presence of bundles). (**b**) To perform the fractal analysis, these images were converted to black and white bitmap images. (**c**) The associated box-counting fractal analysis plots, where the log of the minimum number of boxes, *N*(*ε*), required to cover the network is plotted with box size, ε. The linearity of the plot indicates the network is fractal, and the slope of the best fit line indicates the fractal dimension *D*, indicated for each image shown on the graph. The arrows indicate the expected limits to assess *D*.

To confirm the findings of the simulations, we applied the same fractal analysis to the CNT network’s thin film samples. Their fractal dimensions were found to range from 1.91 to 1.94 (±0.01). Figure 4 shows three example images of thin films analyzed and their resulting fractal box-counting plots. The top film has a density of 3.0 tubes/μm^2^ (±0.5) and is below percolation. The middle and bottom images are from different regions of the same sample, with a density of 9.9 tubes/μm^2^ (±0.9). The average tube lengths determined for the films shown were 1.6, 1.9, and 1.9 μm (±0.5), respectively. The linearity of the plots in Figure 4c indicate a fractal behavior of over 1.5 orders of magnitude. The arrows on Figure 4c indicate the expected observation limits, with the lower limit set by the smallest feature size and the upper limit set by the counting statistics of the box-counting algorithm. In addition to showing the expected fractal character of the networks, our results also indicate that *D* is robust across a given thin film. The middle and bottom rows of Figure 4 are images from two different regions of the same CNT network film, and both are characterized by the same *D* value (1.94 ± 0.01), which is larger than the *D* value of the lower density CNT film shown in the top row of Figure 4.

#### 3.2.2. Electrical Characterization

**Figure 5:**
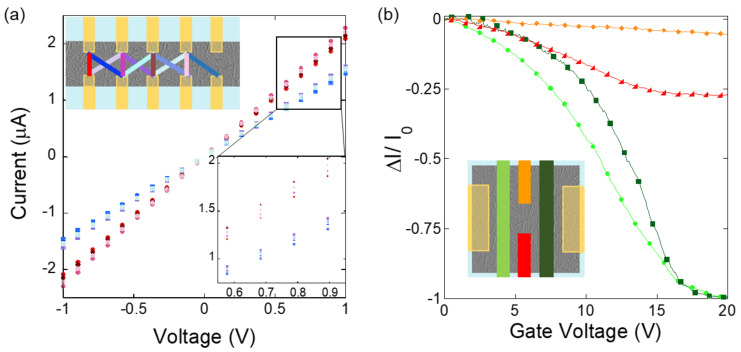
(**a**) Current voltage measurements for ten electrode CNT thin film devices. The device geometry is shown in the inset. The colored lines between electrodes indicate the electrode pairings and are color-coded to associated data in the graph. Vertical electrodes are spaced 20 μm apart, and neighboring electrodes are 30 μm apart from center to center. (**b**) Normalized current change as a function of gate voltage for a different device with two electrodes and four independent top gates. The inset shows the device geometry with gates color coded to match the data, and source and drain electrodes shown in gold. The distance between source and drain electrodes is 60 μm, and the nearest gates from the electrodes is 8 μm. Left and right gates each cover a 60 μm × 10 μm region of the channel, while middle gates each cover a 20 μm × 10 μm region.

To determine the degree to which thin film CNT networks are inhomogeneous we created an open channel CNT network device (see Figure 1a) with ten electrodes spaced along the channel. The inset to Figure 5a shows the device’s geometry. The vertical spacing between device electrodes is 20 μm, and the horizontal spacing between neighbors is 30 μm (center to center). The density of the thin film used in this device was 3.3 tubes/μm^2^ (±0.8), and the average tube length was 1.5 μm (±0.5). Current voltage measurements were made for all possible electrode combinations. In all cases the current voltage characteristics were linear, with well-defined resistances between pairs. This result is important for the proposed neural sensor operation because it allows for a reliable implementation of an established “current library” across different source drain biases. When compared, the resistances of all analogous electrode pairs were discernible, indicating that the network inhomogeneity is measurable.

As an example, Figure 5a shows the current versus voltage behavior measured between all vertical and crossed nearest neighbor electrode pairs. The resistances from all vertical electrode pairings (shades of red) can be readily distinguished from the crossed nearest neighbor electrode pairings (shades of blue). This is expected due to the difference in length between the two sets of electrodes in each case. Furthermore, specific regions of the network of the same electrode-electrode distance were also distinguishable, resulting in a detectable electrical asymmetry which becomes more pronounced at larger source-drain voltages. CNT thin films have the required statistical fractal structure we are looking for to increase the spatial accuracy of neuron detection. This is a key result because it means that the location degeneracy associated with the uniform channel of the simulated sensor in Figure 2 is reduced. As a result, we expect sensors using CNT thin films to have a larger spread of data in current space data (with each point representing the current signatures for a given neuron scenario), allowing for an increased discernment of neurons during sensing.

Another characteristic essential to the implementation of CNT thin films in the proposed sensor is whether the localized electric fields expected from individual neurons firing are enough to measurably impact the current in actual devices. To determine this, we investigated the CNT network’s electrical sensitivity to localized gating. CNT devices fabricated with four top gates (see Figure 1b) of two different sizes were used. The inset of Figure 5b shows the device design, which includes two gates covering 60 × 10 μm^2^ network regions each, and two gates covering 20 × 10 μm^2^ regions each. The sizes of the top gates used are comparable to the neurons and represent the impact that a neural signal would have on the network current.

Top gated devices were fabricated using CNT films of ten different deposition submersion times. We analyzed AFM images of films created under the same circumstances at the same time and verified that CNT density increased with deposition time. Figure 5b shows the normalized current response versus the gate voltage to each of the four top gates from a device with a CNT deposition time of 10 min. Consistent with all devices measured, the network showed a unique current response for each individual top gate. As expected, the larger gates changed the network current the most, but the response was measurable for all gates.

These measurements were made under dry gating. Under these conditions we estimated the electric potential at the CNT network surface to be 1% of the applied gate voltage. This was done using the Poisson equation and solving it numerically (SU8 has a dielectric constant of 3.9). Typical extracellular neural signals range from 0.5 to 1 mV, which corresponds to a relatively small change in current for our devices. However, actual neuronal measurements would be performed under liquid gating conditions. CNT thin film devices have a significant larger network response under liquid gating than dry back gating [15,28]. For example, a normalized current change of 0.5–1 nA can be achieved with a gate potential of 0.5 mV. The exact range can be further altered by controlling the CNT density and the operation potential of the FET [28]. In addition, the measurements in Figure 5b were taken with a source-drain current of 100 mV. Increasing this voltage will increase the distinguishability between changes in current signatures, as seen in Figure 5a. We therefore expect changes in network current induced by neural signals to be observable with our CNT network devices.

Of the devices measured, the device with the lowest density, of 4 tubes/μm^2^, had the greatest sensitivity to gating, and device sensitivity decreased as the density increased. This trend is consistent with what we have seen in CNT thin film aptasensors where devices with sparse network densities, near percolation, performed better at sensing [21]. This result has significant importance for the tuning of CNT thin film properties for enhanced neural sensor performance.

## 4. Discussion

Our simulations and measurements show that randomly aligned CNT networks have the statistical fractal qualities required for the active layer of the proposed neural sensor. Furthermore, our experiments highlight the dependence of the CNT network’s electrical sensitivity on the network’s proximity to the percolation threshold. This provides an opportunity to further tune the system qualities for enhanced sensor performance. To explore the latter effect, we returned to the simulations to investigate the relationship between electrical sensitivity and the fractal dimension *D*.

**Figure 6:**
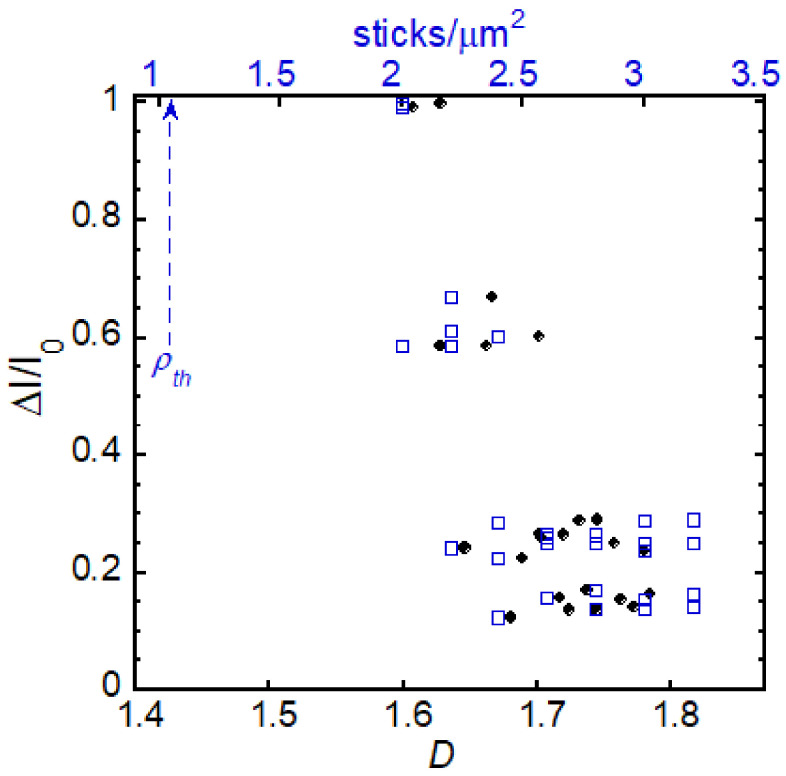
Normalized change in current as a function of fractal dimension, *D*, and CNT density for networks close to the percolation threshold, indicated with the blue dashed arrow.

We simulated a series of CNT networks (with dimensions of 5 μm × 10 μm and tube length of 1.4 μm) with densities within a narrow range, close to the percolation threshold. The percolation threshold for these systems was estimated to be 1.05 tubes/μm^2^. For each of the five different network sets simulated, we generated seven different densities and computed the transfer characteristics. For each network, we determined the *D* value and electrical sensitivity. Figure 6 shows the resulting normalized current change versus *D* (bottom axis) and density (top axis).

Figure 6 is consistent with experimental results showing that network electrical sensitivity dramatically increases as the system approaches percolation [20,21]. Near percolation there are just a few conductive paths making any local changes to the current along the path, such as that from a neuron firing, significant to the total network current. At densities beyond the critical point, adding more tubes creates more paths, and this stabilizes the conductive properties of the network. The evolution of *D* with the addition of tubes parallels the evolution of the conductive properties of the network. As a result, the *D* value is an important indicator of a system’s enhanced electrical sensitivity.

In theory, the best CNT film for sensing would be close to percolation, where sensitivity is at its maximum. However, the variability in the conductive properties of networks very close to the percolation threshold makes them too unreliable as working sensors. For example, when simulating networks in the narrow range just past percolation, the probability that networks have not yet formed a conducting path is more than 95%. For example, 1 in 66 simulated networks with a density of 1.8 sticks/μm^2^ is percolated. This value becomes 1 in 17 at a density of 2 sticks/μm^2^. Fortuitously, Figure 6 shows that beyond the percolation threshold in the range when the *D* value begins to saturate, we see still observe the significant normalized current responses necessary for sensing capacities. We estimate that the device shown in Figure 5b is outside of the region shown in Figure 6 and closer to the region when *D* begins to saturate. While this higher *D* value is not ideal, the thin film still has the essential features from its statistical fractal structure (non-linearity and inhomogeneity) for enhanced sensing.

Before concluding, we note that while the inhomogeneity within a given network structure is an advantage for increasing the spatial accuracy of neuron detection, it introduces a challenge for sensor calibration. Our sensors use a library of data in current space which represents the measured current signature associated with different neuron locations and signal strengths. This library could be calibrated using a controllable metal electrode, which operates as an electrostatic gate to simulate neuron potentials. The gate would be moved to specific locations in the CNT network while the current signature associated with each electrode position is measured to create the calibration library. A 100 μm^2^ device with a 20 μm electrode would require 25 measurements for calibration before operation. Because each CNT thin film of the same density will have significant differences in the arrangement of tubes (as seen in AFM images in Figure 4), each fabricated sensor will likely have a unique library of current signatures. Consequently, each sensor might require individual calibration before operation. However, combining calibration with device imaging could make it possible to reduce the measurements necessary for individual device calibration. For example, using machine learning to identify certain commonalities amongst CNT network features and their resulting libraries, it might therefore be possible to simple image each device to obtain its library.

## 5. Conclusions

We have taken the key initial steps in the application of CNT-based fractal sensing of neural networks. We have used simulations and an experimental characterization of CNT thin films as powerful tools to show that CNT random networks satisfy the criteria for the fractal active layer of our proposed multi-electrode neural sensor. We have also shown that a CNT network’s fractal dimension is linked to its electrical sensitivity. Our next step will be to build the sensor and perform in-vitro investigations of its interaction with neural networks. For these measurements, it will be important to determine the electrical impact that the nutrient medium has on the CNT network conductance. To do this, the current signature of the sensor will be measured for a number of nutrient mixtures in the absence of neurons under experimental conditions similar to those for in-vitro measurements. Variations in these measurements will then provide a noise level for each of the currents in the library and establish a baseline to which neuron network data can be compared. Sensor performance will be assessed by comparing the electrical performance with two photon calcium imaging [33]. Prior to these experiments, it will be important to explore fabrication approaches aimed at lowering the *D* value of the CNT network to the optimal value of 1.65—which will offer increased sensitivity whilst still being sufficiently far from the threshold to fabricate reliably.

## Data Availability

The data presented in this study are openly available in FigShare at https://figshare.com/articles/dataset/Figure_5_data/14156483 (accessed on 4 March 2021).

## Supplementary Materials

The following are available online at https://www.mdpi.com/2079-4991/11/3/636/s1, Figure S1: Raman spectra of CNT thin film.
