# Supplementary material for: Investigation of Fractal Carbon Nanotube Networks for Biophilic Neural Sensing Applications
Source: Nanomaterials (Basel). 2021 Mar 4;11(3):636. doi: 10.3390/nano11030636 (PMC8000135; doi:10.3390/nano11030636)
Supplement: Supplementary file 1 [file nanomaterials-11-00636-s001.pdf]

nanomaterials

MDPI

Supporting Information

Investigation of Fractal Carbon Nanotube Networks for Biophilic Neural Sensing Applications

L.A. Browning [1,2], W. Watterson [3], E. Happe [1,2,4], S. Silva [4], R. Abril Valenzuela [4], J. Smith [3], M.P. Dierkes [4], R.P. Taylor [3], N.O.V. Plank [1,2] and C. A. Marlow [4,*]

1 School of Chemical and Physical Sciences, Victoria University of Wellington, Wellington 6021, New Zealand; leo.browning@vuw.ac.nz (L.A.B.); erica.happe@vuw.ac.nz (E.H.); natalie.plank@vuw.ac.nz (N.O.V.P.)
2 The MacDiarmid Institute for Advanced Materials and Nanotechnology, Victoria University of Wellington, Wellington 6021, New Zealand;
3 Materials Science Institute, Physics Department, University of Oregon, Eugene, OR 97403-1274, USA; william.j.watterson@gmail.com (W.W.); jsmith767@gmail.com (J.S.); rpt@uoregon.edu (R.P.T.)
4 Physics Department, California Polytechnic State University, San Luis Obispo, California, CA 93407, USA; ssilva22@calpoly.edu (S.S.); rabrilvalenzuela@physics.ucsb.edu (R.A.V.); marissadierkes@gmail.com (M.P.D.)
* Correspondence: camarlow@calpoly.edu

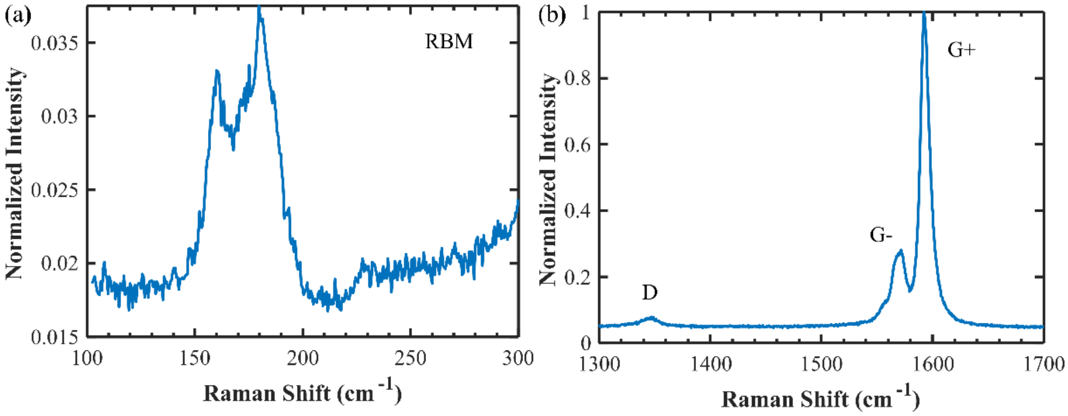

Figure S1. Raman spectra of CNT thin film. Spectra were taken at a laser excitation of 514 nm (Horiba Jobin-Yvon LabRAM HR800). (**a**) Radial breathing mode peaks at 162 cm^{-1} and 180 cm^{-1} corresponding to diameters of 1.5 nm and 1.4 nm, respectively. These results are consistent with the documentation provided by the manufacturer, NanoIntegris, which indicates that the single-walled CNTs used consist of 99% semiconducting CNTs with diameters ranging from 1.2 to 1.7 nm with a mean diameter of 1.4 nm. (**b**) The D-band feature at 1340 cm^{-1} is much smaller than G+ peak at 1590 cm^{-1} and the G- peak at 1570 cm^{-1} indicating minimal defects in the CNTs.
